# β-catenin, Twist and Snail: Transcriptional regulation of EMT in smokers and COPD, and relation to airflow obstruction

**DOI:** 10.1038/s41598-017-11375-x

**Published:** 2017-09-07

**Authors:** Malik Quasir Mahmood, Eugene Haydn Walters, Shakti D. Shukla, Steve Weston, Hans Konrad Muller, Chris Ward, Sukhwinder Singh Sohal

**Affiliations:** 1 NHMRC Centre of Research Excellence for Chronic Respiratory Disease, School of Medicine, University of Tasmania, Hobart, Tasmania 7000 Australia; 2 Institute of Cellular Medicine, Newcastle University, Newcastle upon Tyne, Tyne and Wear UK; 3 School of Health Sciences, Faculty of Health, University of Tasmania, Launceston, TAS 7248 Australia

## Abstract

COPD is characterised by poorly reversible airflow obstruction usually due to cigarette smoking. The transcription factor clusters of β-catenin/Snail1/Twist has been implicated in the process of epithelial mesenchymal transition (EMT), an intermediate between smoking and airway fibrosis, and indeed lung cancer. We have investigated expression of these transcription factors and their “cellular localization” in bronchoscopic airway biopsies from patients with COPD, and in smoking and non-smoking controls. An immune-histochemical study compared cellular protein expression of β-catenin, Snail1 and Twist, in these subject groups in 3 large airways compartment: epithelium (basal region), reticular basement membrane (Rbm) and underlying lamina propria (LP). β-catenin and Snail1 expression was generally high in all subjects throughout the airway wall with marked cytoplasmic to nuclear shift in COPD (P < 0.01). Twist expression was generalised in the epithelium in normal but become more basal and nuclear with smoking (P < 0.05). In addition, β-catenin and Snail1 expression, and to lesser extent of Twist, was related to airflow obstruction and to expression of a canonical EMT biomarker (S100A4). The β-catenin-Snail1-Twist transcription factor cluster is up-regulated and nuclear translocated in smokers and COPD, and their expression is closely related to both EMT activity and airway obstruction.

## Introduction

Chronic obstructive pulmonary disease (COPD) is mainly smoking-related and primarily reflects small airway fibrosis and destruction with later development of emphysema in some. Approximately 50% of smokers develop COPD eventually^1, 2^ and probably significantly more if specific small airway measurements are used^3^ for assessment. COPD is also strongly related to lung cancer development^4–6^.

We and others have described active EMT in the airway epithelium smokers and especially in COPD^7–9^. This is likely to be related to epithelial basal stem cell reprogramming^10^. There is some work, mainly in epithelial cell culture on the mechanisms of EMT in the airways^11^. But we also recently reported evidence in airway biopsies for involvement of TGF-β1 and its Smad transcription factor system^12^.

β-catenin is physiologically part of a major cell-surface adhesion complex^13^ but if released from there becomes part of another important pro-pathological transcriptional factor cascade. This is classically induced by Wnt (Wingless tail) ligand activating the Frizzled cell surface receptor^14–16^. We hypthesise that β-catenin may be involved in airway EMT activity, following a recent report of increased Wnt expression in lung epithelial cells from COPD patients^17^.

Binding of Wnt ligand to the Frizzled receptor induces its phosphorylation and through this inactivation of the cytoplasmic β-catenin sequester, glycogen synthase kinase-3β(GSK-3β)^18^. This contributes to cytosolic accumulation of β-catenin and then nuclear translocation where it binds with lymphoid enhancing binding factor (LEF-1). In addition, TGF-β1 can signal to increase LEF-1 expression through SMADs and also to inhibit GSK-3β^19, 20^.

Snail1, is a zinc finger binding transcription factor, repressing transcription of membrane adhesions, so releasing β-catenin^21, 22^. Reciprocally, the Wnt-β-catenin and PI3K-AKT mechanism also increase Snail1 activity by preventing its phosphorylation by GSK-3β, which enhances EMT^23^. A mutual interaction also seems to exist between SMADs and Snail1 for induction of EMT^24, 25^.

Twist is a basic helix-loop-helix transcription factor which also plays a key role in EMT progression^26^. As with Snail1, Twist expression down-regulates epithelial gene expression and activates mesenchymal gene expression^27^. Twist activity intracellularly is augmented by its phosphorylation by mitogen activated protein kinase (MAPK)^28^.

The role of this key EMT-related transcriptional factor cluster (β-catenin, Snail1 and Twist) specifically in development and progression of airway remodelling in smokers/COPD is still largely unexplored, but our hypothesis is that this system is intimately involved in EMT activity in the airways and its down-stream pathophysiological consequences, as well as having cross-relationship with the SMAD pathway system. Thus, the present study has evaluated their protein expression and cellular compartmentalisation in airway biopsies from COPD subjects and appropriate controls. We have also explored relationships between these transcriptional factors and EMT activity (represented by the mesenchymal marker S100A4), with TGFβ1 and SMADs, and finally with airflow obstruction as the final functional outcome of these complex processes.

## Results

### Large airway

#### Basal cells

**Figure 1:**
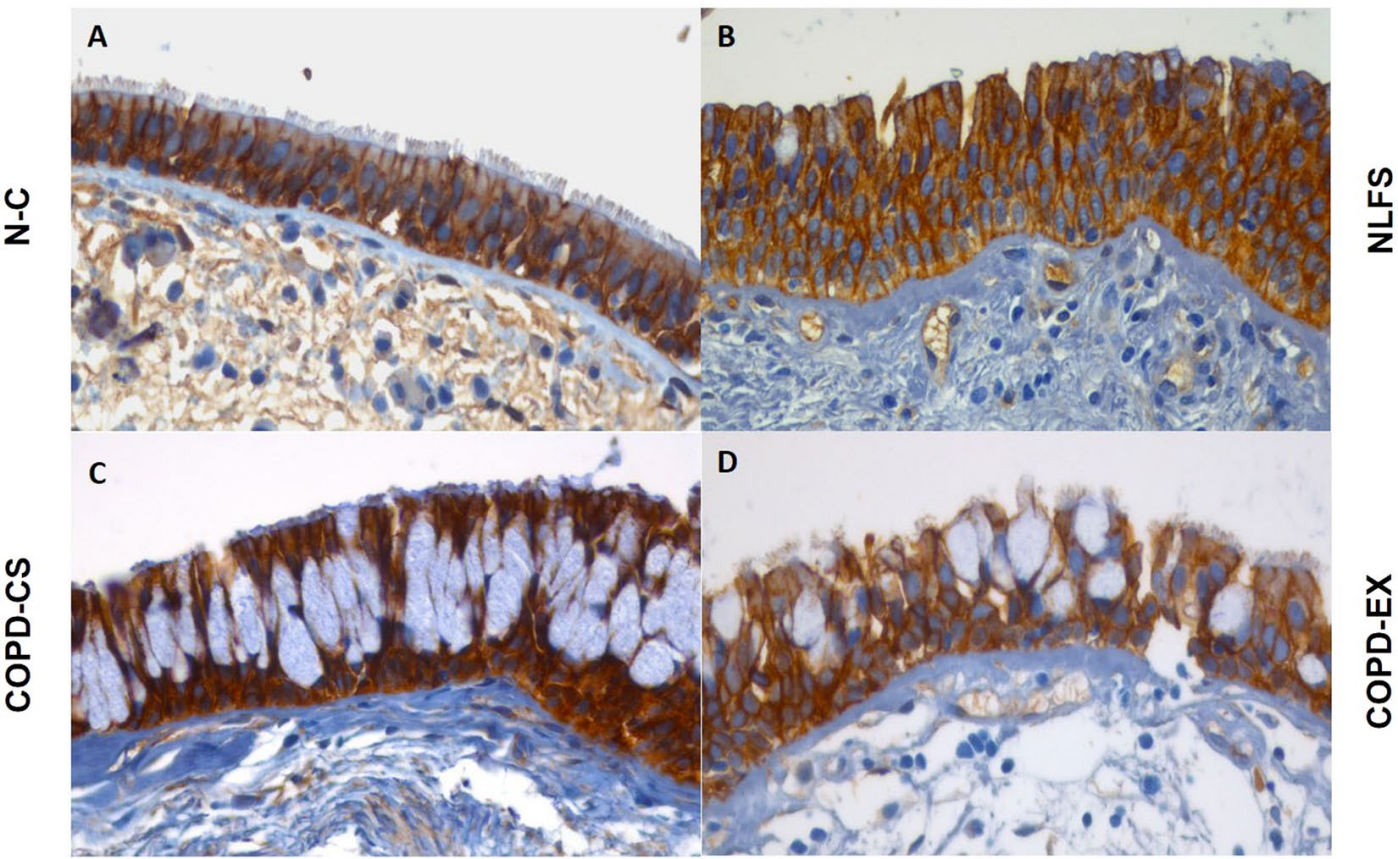
Representative photomicrograph of β-catenin expression in 4 different study groups: (**A**) healthy non-smokers (N-C), (**B**) smokers with normal lung function (NLFS), (**C**) current smoking COPD (COPD-CS) and (**D**) ex-smokers with COPD (COPD-EX). Original magnification, ×400. Scale bar = 50 µm.

β-catenin: There was general staining for β-catenin in the basal cells of airway epithelium in all groups with no difference in % of cells expressing it. However, there were striking change in the cellular distribution of β-catenin from cell membrane to cytoplasm in normal lung function smokers (NLFS) and COPD-Ex, but also to the nucleus in COPD current smokers (COPD-CS). The ratio of number of cells with predominant nuclear rather than cytoplasmic predominance was significantly higher in COPD-CS in comparison to both normal control (NC) and NLFS (P < 0.01) with COPD-Ex being intermediate (Figs 1 and 4).

**Figure 2:**
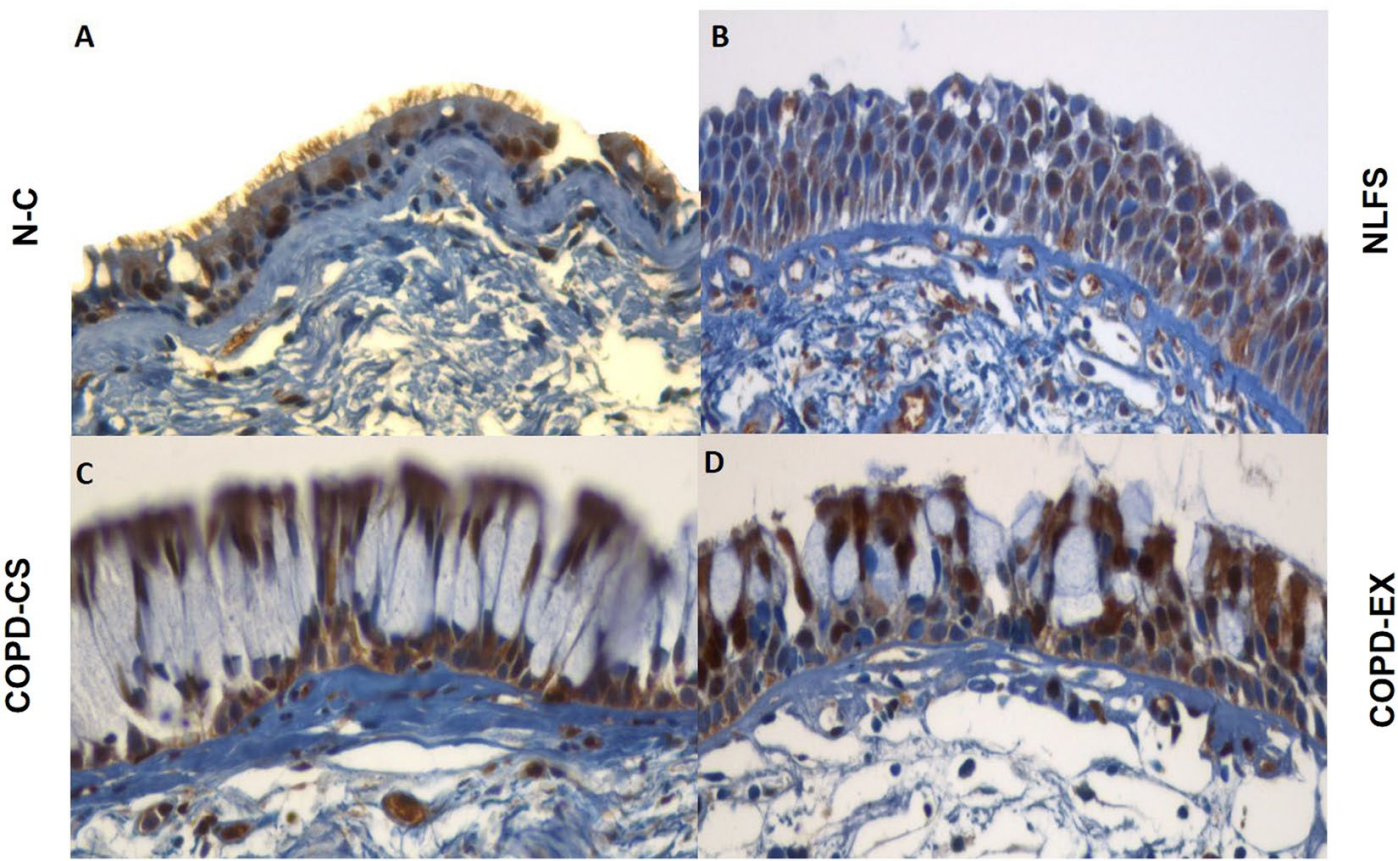
Photomicrographs of Snail1 expression representing 4 different study groups: (**A**) healthy non-smokers (N-C), (**B**) smokers with normal lung function (NLFS), (**C**) current smoking COPD (COPD-CS) and (**D**) ex-smokers with COPD (COPD-EX). Original magnification, ×400. Scale bar = 50 µm.

Snail1: Snail1 expression was less uniform among basal cells, but there was a significant increase in percent cells expression in both NLFS and COPD-CS (P < 0.05). In addition, there was again a significant shift from cytoplasmic to nuclear expression only in the COPD-CS group (P < 0.01) though with an intermediate change from NC, in the NLFS and COPD-Ex groups (P < 0.01) (Figs 2 and 4).

**Figure 3:**
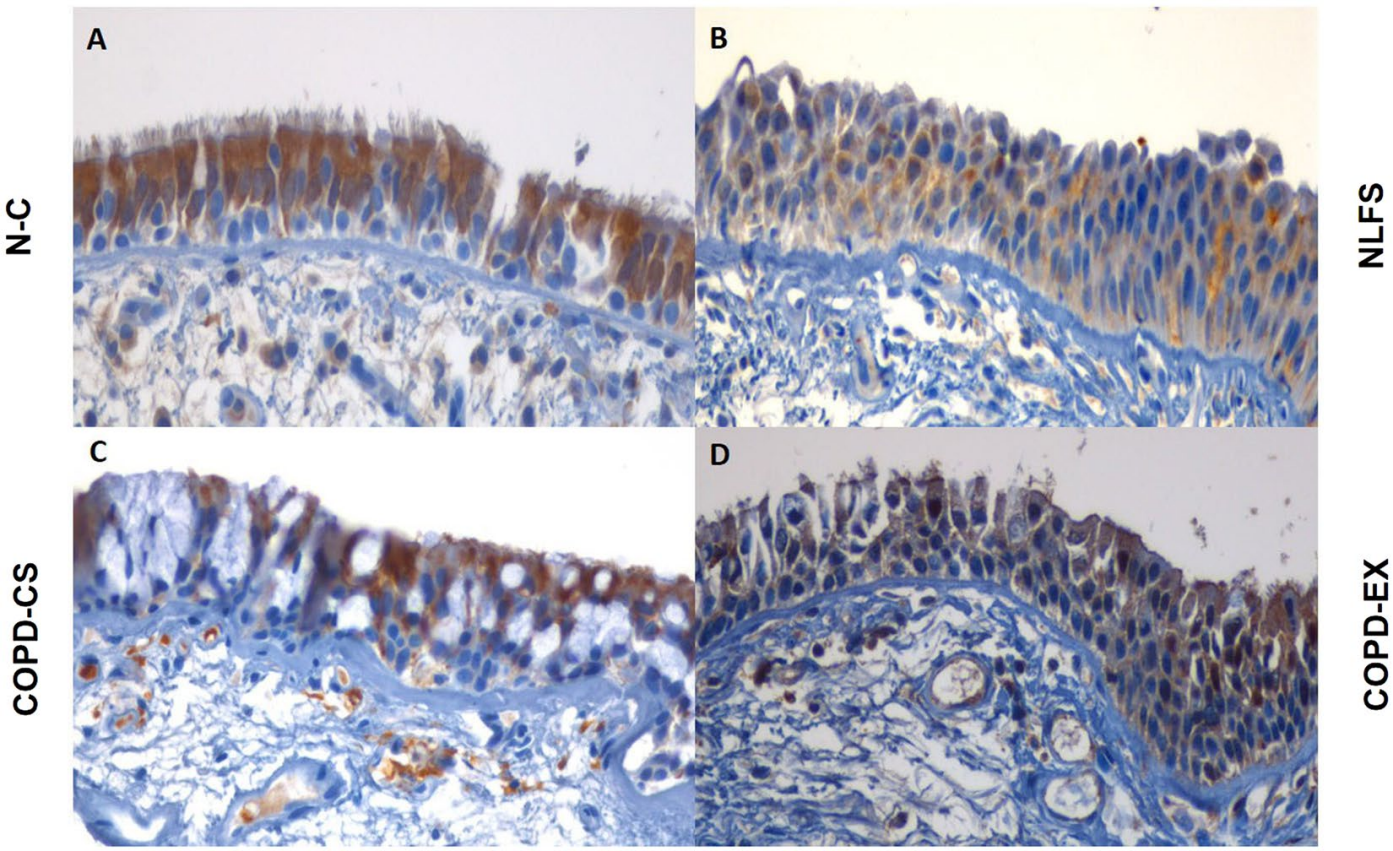
Representative photomicrograph of Twist expression in 4 different study groups: (**A**) healthy non-smokers (N-C), (**B**) smokers with normal lung function (NLFS), (**C**) current smoking COPD (COPD-CS) and (**D**) ex-smokers with COPD (COPD-EX). Original magnification, ×400. Scale bar = 50 µm.

**Figure 4:**
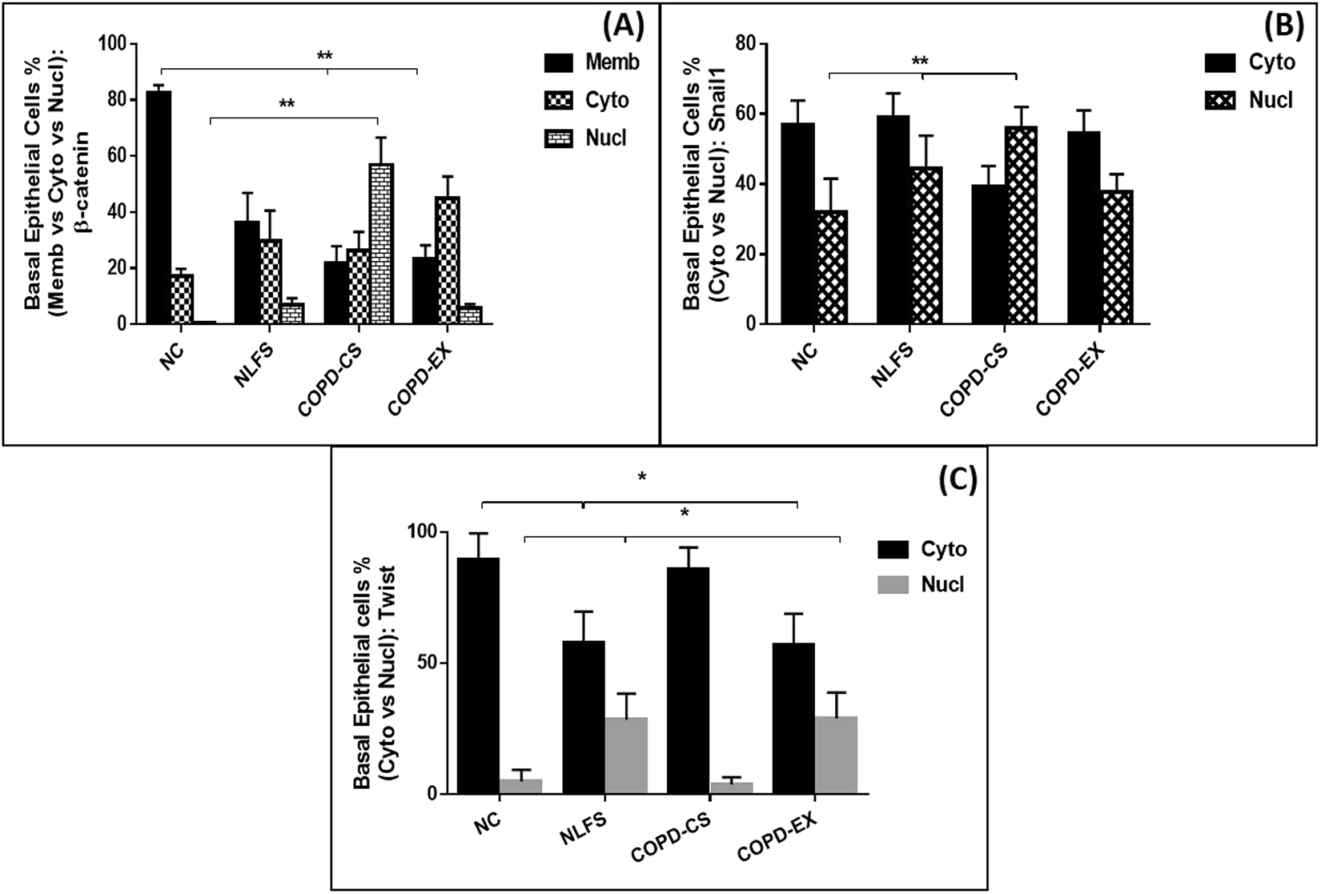
Cytoplasmic versus nuclear % basal cells expressing: (**A**) β-catenin (**B**) Snail1 and (**C**) Twist; comparing healthy non-smokers (N-C) n = 15, smokers with normal lung function (NLFS) n = 15, current smoking COPD (COPD-CS) n = 20 and ex-smokers with COPD (COPD-EX) n = 15.

Twist: Twist expression was prominent in the NC group but only in the more apical cell areas. In contrast, there was more basal cell expression in all other groups (P < 0.01). There was shift from the cytoplasmic to nuclear compartment expression only in the 2 groups, NLFS and COPD-Ex (P < 0.05), and not in COPD- CS (Figs 3 and 4).

#### Rbm cells

**Figure 5:**
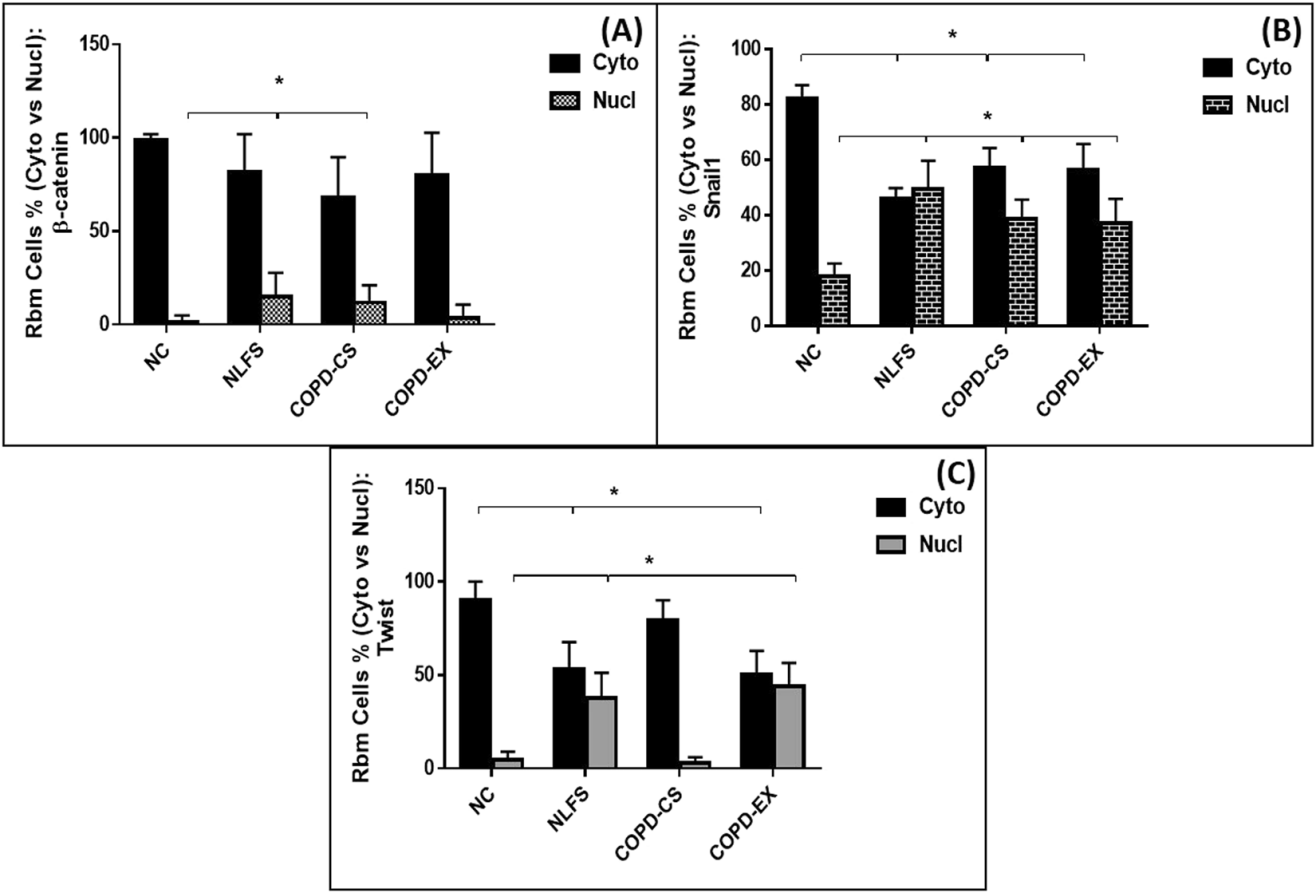
Cytoplasmic versus nuclear % Rbm cells expressing: (**A**) β-catenin (**B**) Snail1 and (**C**) Twist; comparing healthy non-smokers (N-C) n = 15, smokers with normal lung function (NLFS) n = 15, current smoking COPD (COPD-CS) n = 20 and ex-smokers with COPD (COPD-EX) n = 15.

β-catenin: There was little difference in general cellular staining between the 4 study groups, but a small though significant shift from cytoplasmic to nuclear expression in both NLFS and COPD-CS(P < 0.05) (Figs 1 and 5), with corresponding increase in nuclear to cytoplasmic ratios (P < 0.05), i.e. a smoking effect only.

Snail1: There was similar and substantial staining of Snail1 in Rbm cells in all 4 groups, but with a marked shift from cytoplasmic to nuclear expression in the 3 clinical groups compared to NC (P < 0.05) (Figs 2 and 5), reflected again in increased nuclear to cytoplasmic ratio for these groups (P < 0.05), i.e. again more of a smoking than COPD effect.

Twist: There was increased expression of Twist in NLFS as compared NC subjects with a small but significant shift towards nuclear expression in both NLFS and COPD-Ex groups (P < 0.05) (Figs 3 and 5) (also P < 0.05 for the corresponding change in nuclear to cytoplasmic ratio). There was little change in the COPD-CS group.

#### LP cells

**Figure 6:**
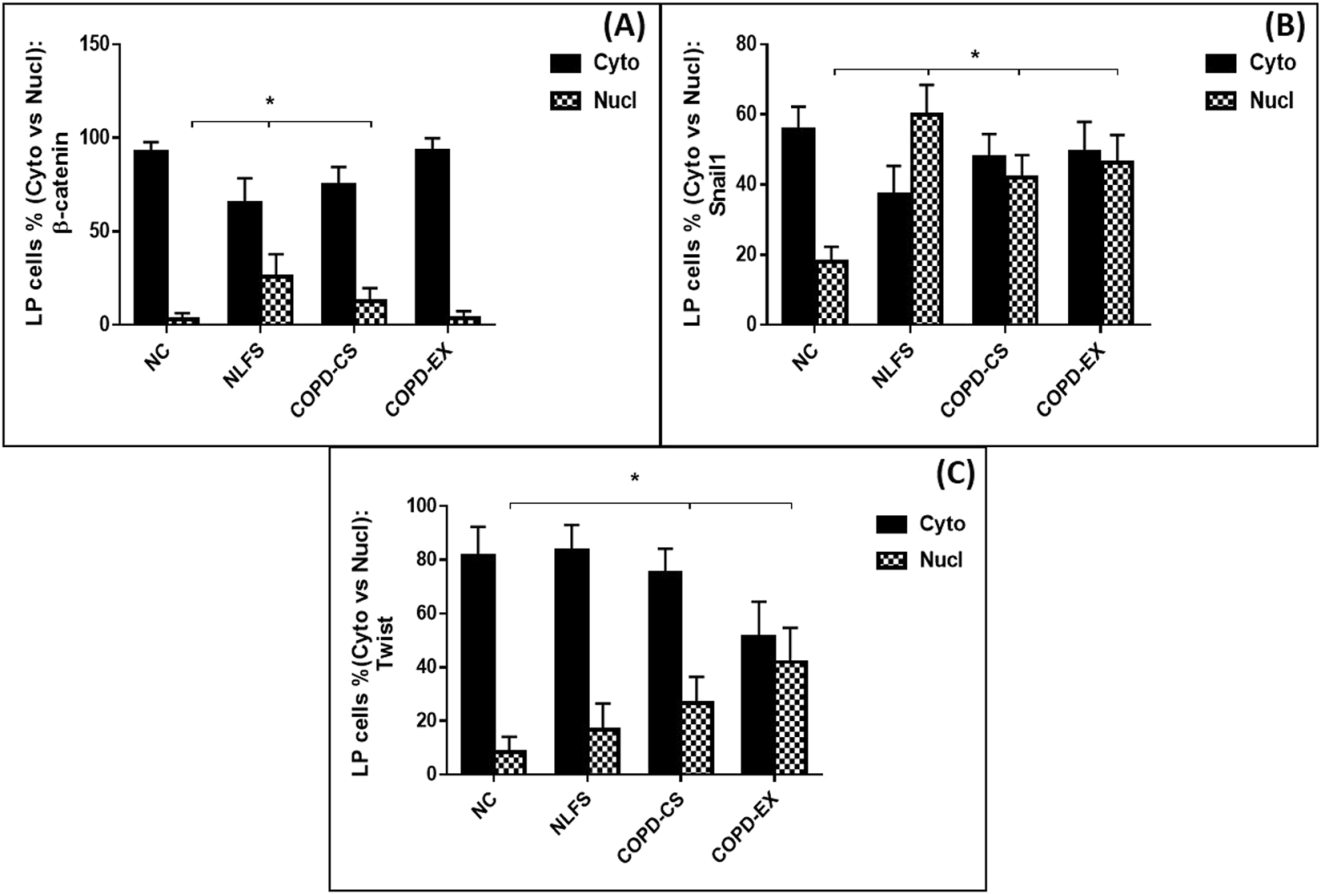
Cytoplasmic versus nuclear % LP cells stained with: (**A**) β-catenin (**B**) Snail1 and (**C**) Twist; comparing healthy non-smokers (N-C) n = 15, smokers with normal lung function (NLFS) n = 15, current smoking COPD (COPD-CS) n = 20 and ex-smokers with COPD (COPD-EX) n = 15.

β-catenin: Matrix staining for β-catenin was observed in NC only, and was absent in all clinical groups, suggesting some change in physico-chemical interactions with matrix proteins. Among LP cells, only approximately 20% of them expressing β-catenin (and the other transcription factors) in each group, but there was a shift in these cells in β-catenin staining to nuclear expression in both NLFS and COPD-CS compared to NC (P < 0.05) (Figs 1 and 6). Similarly, nuclear to cytoplasmic ratio was also significantly higher in NLFS and COPD-CS (P < 0.05).

Snail1: No matrix staining was observed in any group. There was again a marked shift from cytoplasmic to nuclear cellular expression in the 3 clinical groups, but especially in NLFS and COPD-CS (P < 0.05) (Figs 2 and 6), with a corresponding shift observed in nuclear to cytoplasmic ratio in NLFS and COPD-CS (P < 0.05).

Twist: Matrix was devoid of Twist staining in the LP in all groups. There was no difference in percent cell staining between groups with a shift from cytoplasmic to nuclear staining only in the COPD groups, especially in COPD-Ex smokers (P < 0.05) (Figs 3 and 6). In addition, nuclear to cytoplasmic ratio was also observed to be high in these COPD clinical groups (P < 0.05).

### Regression Analyses

**Figure 7:**
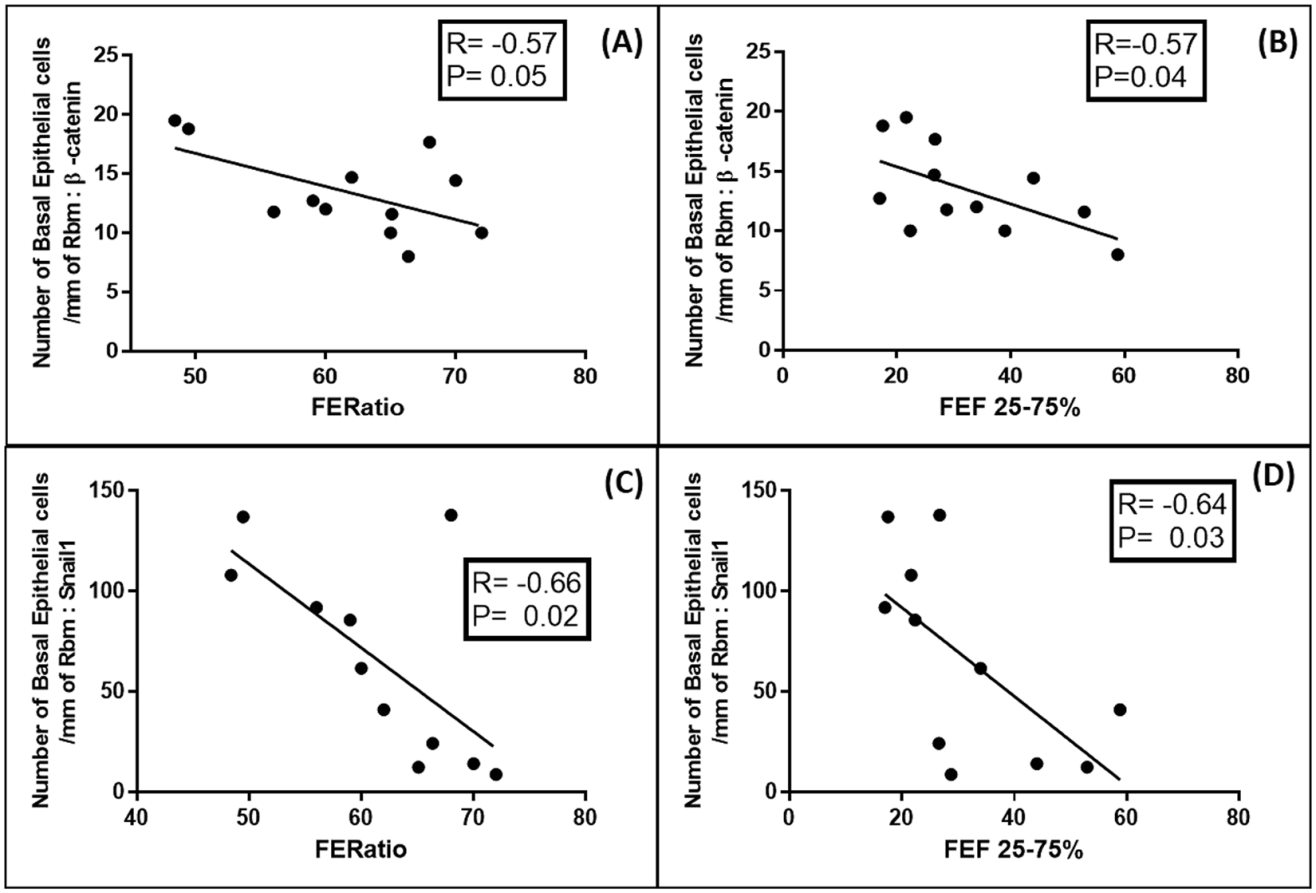
Regression analyses for the current-smoking COPD group (COPD-CS) n = 12: (**A**) Correlation between number of basal epithelial cells per mm of Rbm, positive for β-catenin and forced expiratory ratio (FER); (**B**) The same for % predicted FEF25-75 (an index of small airway calibre); (**C**) Correlation between Snail1 positive basal epithelial cells and forced expiratory ratio (FER); (**D**) Correlation between basal epithelial cells positive for Snail1 with % predicted FEF25-75.

**Figure 8:**
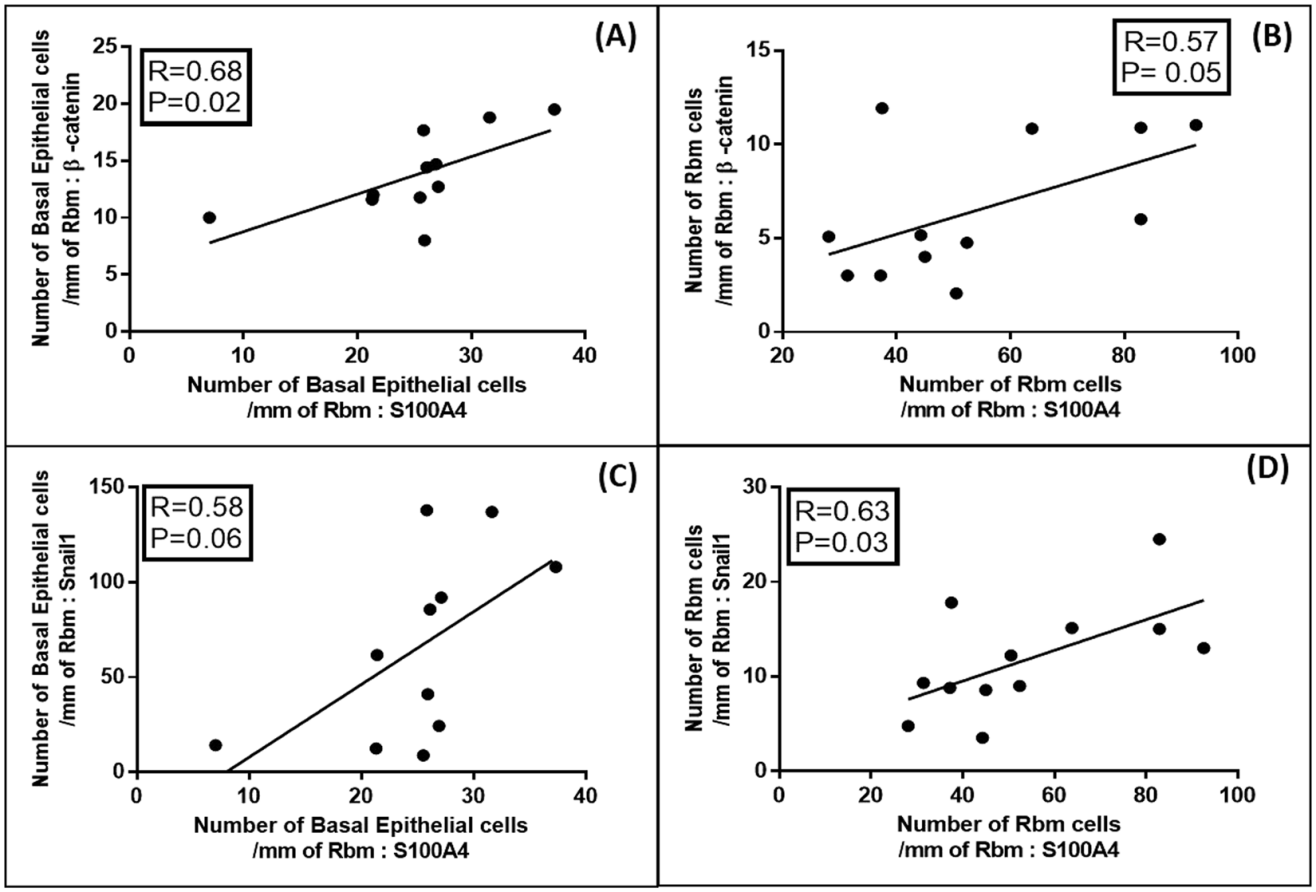
Regression analyses for the current-smoking COPD group (COPD-CS) n = 12: (**A**) Correlation between number of basal epithelial cells per mm of Rbm, positive for β-catenin with S100A4 positive basal epithelial cells; (**B**) Correlation between number of Rbm cells per mm of Rbm, expressing β-catenin with S100A4 positive Rbm cells; (**C**) Correlation between number of basal epithelial cells per mm of Rbm, positive for Snail1 with S100A4 positive basal cells; (**D**) Correlation between Rbm cells expressing Snail1 with S100A4 positive Rbm cells.

**Figure 9:**
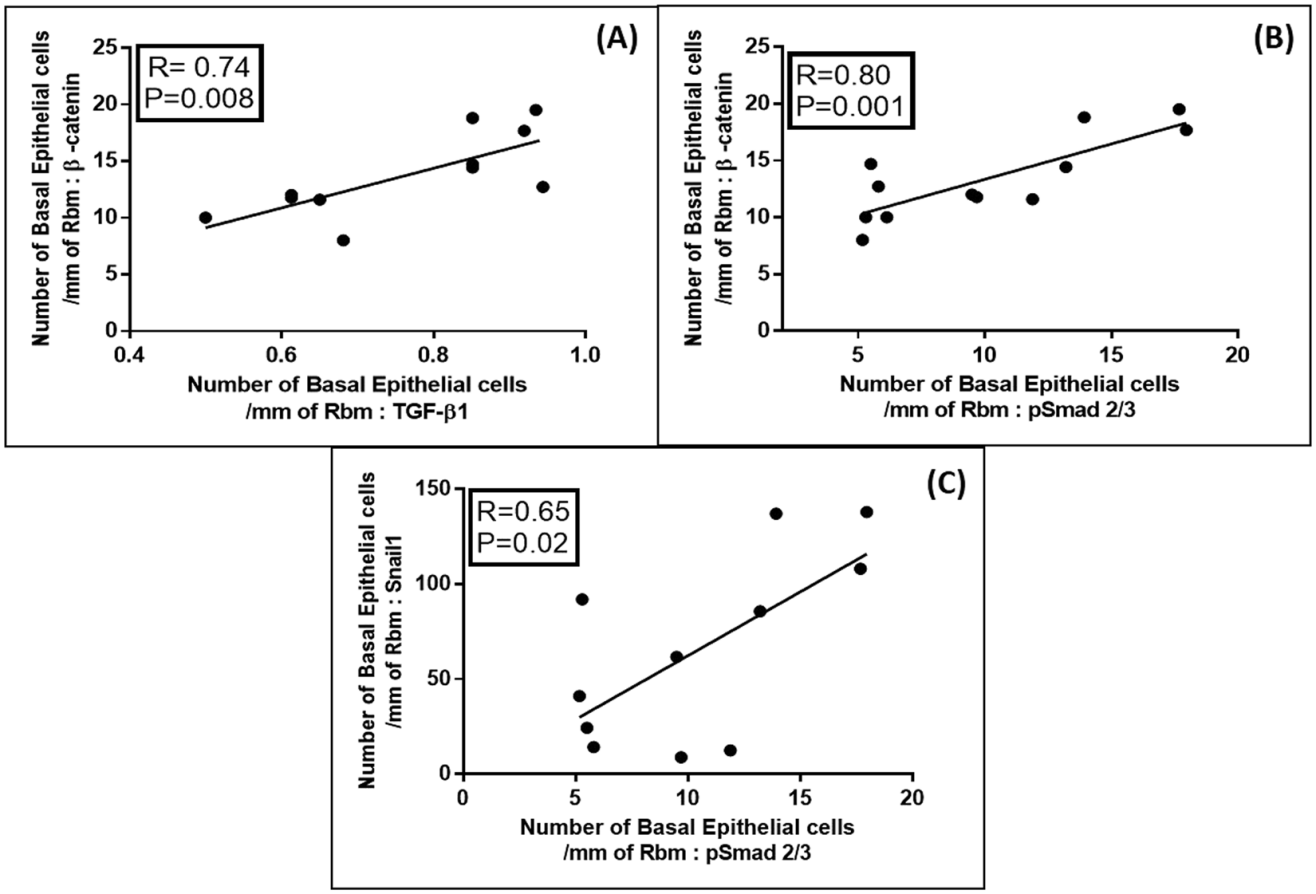
Regression analyses for the current-smoking COPD group (COPD-CS) n = 12: (**A**) Correlation between number of β-catenin positive basal epithelial cells per mm of Rbm, with TGFβ1 positive basal epithelial cells; (**B**) Correlation between number of basal epithelial cells positive for β-catenin with pSmad 2/3 positive basal epithelial cells; (**C**) Correlation between basal epithelial cells expressing Snail1 with pSmad 2/3 positive basal epithelial cells.

The relationships between β-catenin and Snail1 cell expressions, each independently with lung function, EMT activity (expressed by S100A4 expression) and TGFβ1-Smad pathway expression, were quite similar in all compartments (i.e. for basal cells, Rbm cells and LP cells). Relationship for Twist were weaker though still generally significant. In general, higher the expressions of the transcription factors, greater was EMT activity and also greater were the levels of airflow obstruction. These relationships were strongest for basal cells data and in COPD-CS (Figs 7–9). We have limited reporting the actual regressions to this specific clinical group because of their visually obvious as well as statistical strength and strategic importance.

### Small airway

**Figure 10:**
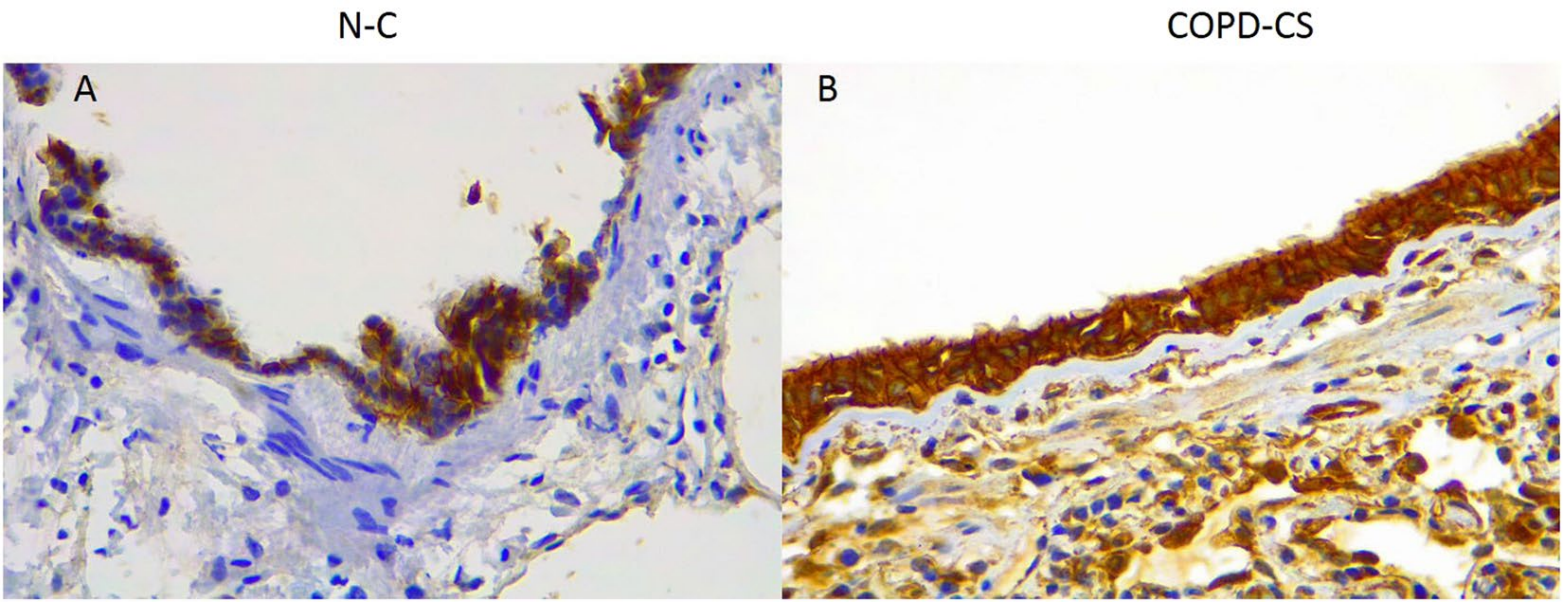
Representative photomicrograph of β-catenin expression in small airway of (**A**) healthy non-smokers (N-C), (**B**) current smoker COPD (COPD-CS). β-catenin cellular staining is more abundant in COPD-CS subjects in the epithelium, but also in the Rbm and sub-epithelial lamina propria. The cellular localisation also changes from a membrane association only, to include the cytoplasmic and nuclear compartment, though difficult to visually differentiate at this magnification which is needed for “photo-micrographs”.

For comparative purpose, we have also stained for β-catenin expression in a small number of lung resection small airway samples from normal and COPD current smoking (COPD-CS). Although data are descriptive only, there was consistently heavier cellular staining in all compartments (i.e. epithelial cells, Rbm and LP cells) in the COPD small airways compared to normal. There was also marked shift from cytoplasmic to nuclear expression in the COPD-CS group (Fig. 10).

### Large airway epithelial E-Cadherin expression

**Figure 11:**
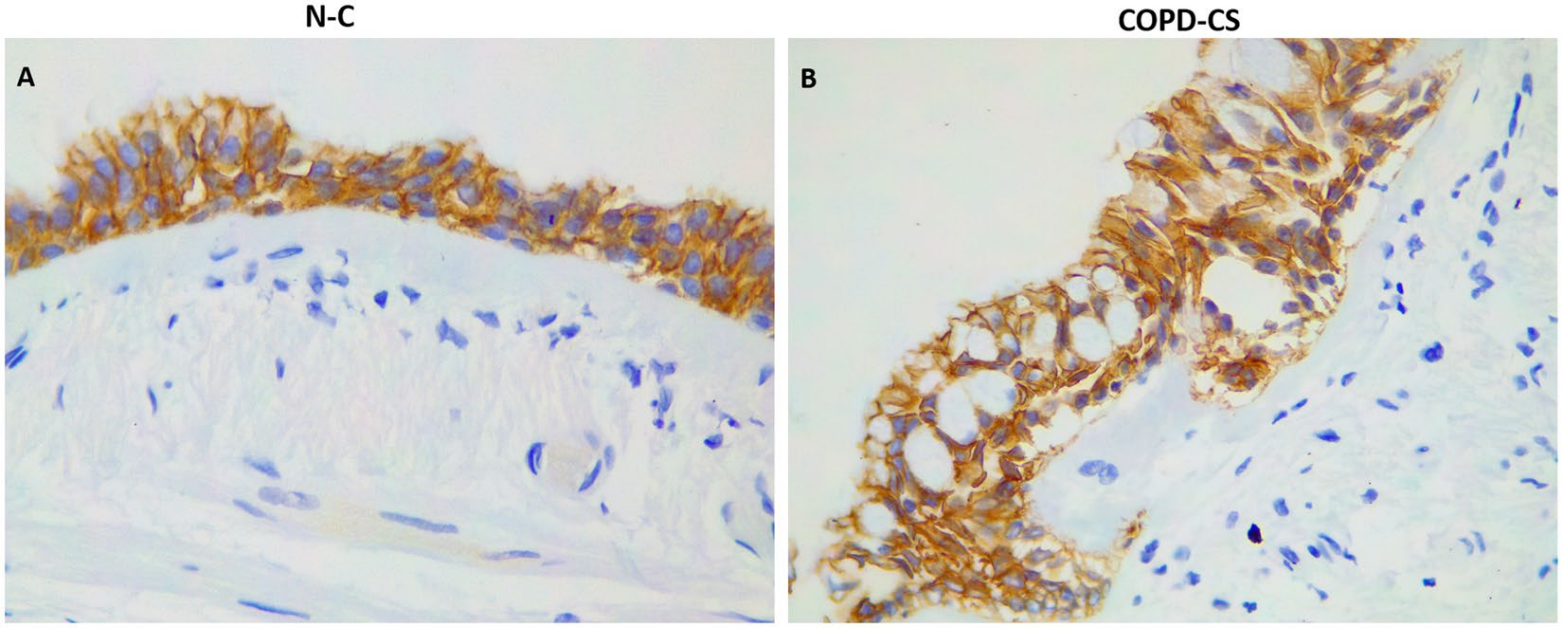
Representative photomicrograph of E-cadherin expression in large airway of (**A**) healthy normal control non-smokers (N-C), and (**B**) current smoker COPD (COPD-CS). E-cadherin staining does not seems to hugely different between the two groups, although a slight decrease is likely, especially circumferentially, in basal epithelium cell expression in COPD-CS.

Down-regulation of E-cadherin in conjunction to changes in β-catenin expression is an important step in EMT. Reduced expression of E-cadherin in COPD airways has been reported before by Gohy *et al*.^11^, Oldenburger *et al*.^29^ and Milara *et al*.^9^. With nuclear localisation of β-catenin in large airway epithelium, we further explored E-cadherin expression in epithelium. We stained for E-cadherin in a small number of large airway samples from normal controls (N-C) and COPD current smoking (COPD-CS). Although data at this stage are preliminary and essentially descriptive only, there was quite strong suggestion of lower expression of E-cadherin in basal epithelial cells in COPD, especially at the margins of the cells (Fig. 11). However, overall, E-cadherin expression was quite high in the whole epithelium, which makes the signal-to-noise ratio overall quite weak.

## Discussion

Smoking related COPD is a major disease of global significance. Its obstructive pathophysiology is predominantly due to small airways fibrosis and progressive luminal obliteration^30^. Some individuals go on to develop emphysema^31^ and many also lung cancer, which should be regarded as part of the COPD^32, 33^. Although we do not yet fully understand the fundamental pathobiology of the core airway components of COPD, including widespread epithelial structural remodelling, it is most likely related to “reprogramming” of basal epithelial (stem) cells^10^. We have described active EMT throughout the airway tree in smokers and especially in COPD^7, 8, 34^, as a significant manifestation of this epithelial basal cell dysfunction^35^ and potentially a gateway to both airway fibrosis and lung cancer^6, 33, 36, 37^.

We have recently published on the TGF-β1-Smad pathway in COPD, and its relationships with both EMT and airway obstruction^12^. We have now addressed similar issues with the classic pro-EMT β-catenin-Snail1-Twist transcription factor cluster, usually thought to be activated by the Wnt system through cell surface Frizzled receptors^18^, but as outlined in the introduction the regulation of the system is complex. However, we have now provided strong evidence for a general up-regulation of this system in smokers. In the basal epithelial cells, this up-regulation was even greater in COPD current smokers, though this was not so marked in Rbm and LP where changes were more of a smoking effect only. It suggests that smoking does activate EMT even without full-blown COPD, which might explain cancer development and some small airway disease even in “normal” smokers. However, in general these processes are more aggressive in COPD itself. Although the evidence is circumstantial at the moment, it seems likely that those “normal” individuals with the highest EMT signal in the airway will be those who go on to get full-blown COPD and to be most likely to develop lung cancer. Ideally, a study should be done to follow a statistically large enough number of normal lung function smokers over several years after initial bronchoscopy, endobronchial biopsy and tissue analysis, and then see who goes on to develop COPD with small airway fibrosis and/or lung cancer. Such a study will be highly informative but logistically very hard and very expensive to undertake.

A particular feature of these up-regulated transcription factor expressions from the cell cytoplasm (and in the case of β-catenin first from the cell membrane) into the cell nucleus. This was evident not only in the epithelial basal cells but also in the hyper-cellular Rbm and to an extent in lamina propria stromal cells. There were strong generalised associations between the expression of these transcription factors and EMT activity (using expression of the classic mesenchymal marker S100A4), and notably also airflow obstruction.

The E-Cadherin/β-catenin complex plays an important role in maintaining epithelial cell integrity, and disrupting this complex at cell margins affects not only the adhesive properties of the epithelium, but the Wnt-signaling pathway as well. In general, aberrant expression of this cell-surface complex is associated with a wide variety of epithelial malignancies and fibrotic pathologies resulting mainly from EMT. In large airways, we have undertaken some preliminary work showing likely decrease in epithelial E-cadherin expression but only in basal epithelial cells of COPD current smokers. However, this is not easy to quantitate, as E-cadherin is very abundant at a tissue level in the whole epithelium, which makes the basal cell signal quite weak in the context of the whole tissue, and much weaker than the β-catenin signal of down-regulation and nuclear transitioning. In a previous publication Gohy *et al*. showed a stronger signal for E-Cadherin reduction in COPD epithelium than in our current observations^11^; this difference may be methodological but we are as one in suggesting active EMT in this tissue.

Although several studies have demonstrated a role of the canonical Wnt/β-catenin signalling pathway in fibrosis and tissue remodelling^38–40^, little is known about how β-catenin may be involved in the pathology of COPD. Our observations do consolidate the recent report of Wnt up-regulation in COPD airways and its induction in cultured airway cells by cigarette smoke^17^. In addition, various growth factors, including TGF-β1, can activate β-catenin signalling either directly or via autocrine Wnt ligand production^38, 41, 42^ and this would fit with our observation of a significant cross-association with the TGF-β1 and SMAD2/3 pathways in these human tissue studies.

Stabilized (non-phosphorylated) β-catenin activates several targe genes including matrix metalloproteinases (MMP’s), growth factors, extracellular matrix (ECM) proteins and pro-inflammatory mediators and enzyme^43–45^ and most importantly has been postulated as a key inducer of EMT in several tissues^46, 47^. In addition, Baarsma *et al*. observed that β-catenin expression is higher than normal in primary pulmonary fibroblast from COPD subjects^48^, which may reflect our findings in LP stromal cells.

Snail1 and Twist have been implicated previously in induction of EMT in COPD with protein and mRNA expression of mesenchymal markers and EMT-related transcription factors increased in cultured epithelial cells^49^. Our findings are novel in that we have not only shown a general increase in expression of transcriptional factors in all airway wall compartments, but also a downstream cytoplasmic to nuclear shift in smokers and especially COPD. These changes were most evident in the basal epithelial cells in current smoker COPD. We suggest that this picture represents a fundamental key to understanding COPD pathogenesis. Most of our data presented here have come from endo-bronchial biopsies of large airways, but the limited data we have presented from small airway samples would suggest that the same key changes are present in COPD in the small airways where pathogenic airway fibrosis and obstruction is greatest.

β-catenin, Snail1 and Twist are intimately interlinked because the majority of β-catenin’s action is mediated through the other two transcription factors^50, 51^. Twist as an important transcriptional factor for fibrosis was first demonstrated in a murine model of virus-induced lung fibrosis and in alveolar epithelial cells of idiopathic pulmonary fibrosis (IPF) patients^52^. Our data suggest that these processes are also active in the airways in smoking-related COPD.

Thus, one of the most remarkable findings in our current study was the significant correlation between basal epithelial cell transcription factor expression with both an EMT activity marker and also airflow obstruction. This latter “mechanistic” relationship gives our findings clinical and potentially translational relevance. However, we also found a strong relationship in our previous study between SMAD expression in the epithelium and airway obstruction^12^ though interestingly this was not found for TGF-β1 expression. This emphasises that factors other than this specific growth factor are also likely to be involved in driving EMT and down-stream airway fibrosis, making this a complex system unlikely to be amenable to a simple therapeutic intervention. Even so, the fact that there is a strong relationship between the β-catenin-Snail1-Twist transcription factor cluster and the previously studied Smad pathway suggests that TGF-activin drivers may be dominant.

The strengths of the present study include the use of relevant human tissue in well phenotyped individuals including mild to moderate COPD patients and comprehensive appropriate controls, and with fairly robust numbers giving sufficient power to detect these fascinating findings. We have focused on mild to moderate COPD patients, because active pathogenic mechanisms at this stage will be core ones, and not unduly influenced by later secondary complications such as infection leading to inflammation and immune activation in the airway lumen and without significant emphysema to affect airflow.

There are also some limitations to this study. Firstly, it is cross-sectional at a single time point and without the potential strength of longitudinal studies which could related variable transcriptional factors expression to individual disease progression. It is noteworthy, for example, that even in the normal smoker control group (NLFS) there were quite strong relationships between transcription factors and decreasing lung function, even if technically all within the non-COPD range, suggesting that there are individuals on the way to full-blown COPD; one could also speculate that they may be of particular risk of lung cancer. Secondly, we are not sure about the detailed phenotype of the of LP cells expressing β-catenin/Snail1/Twist, although descriptively they seemed to be stromal cells and not immune/inflammatory. Double staining will be a future goal. Thirdly, our control subjects were somewhat younger than the smoker/COPD group, but transcription factor expression levels were not age related in any group; and finally, this study used many large airway biopsies while the predominant anatomic site of airflow limitation is in the small airways. We did this because recruiting volunteers for bronchoscopic airway sampling allowed us access to physically fit subjects with well-defined phenotypes including COPD subjects with relatively mild disease and without confounding drug treatment or the pathology present in resected lung tissues. Further, we know that although EMT is especially active in larger airways it is also present in small airways and lessons learned in one site is likely to reflect also what is happening throughout the airway tree; it is telling that both EMT activity^7, 8, 34^ and these current transcription factor expressions were strongly related to small airway function (FEF75-25%). Even so, it is now certainly well worth the effort to try and replicate these finding comprehensively in small airway samples. Our preliminary data from small airways presented here suggest that this will be well worthwhile.

Our general goal in this program over several years has been to comprehensively define the key underlying pathology in COPD airways, and latterly to define main drivers and transcriptional pathways that contribute to what we believe is a core part of the COPD end-phenotype, namely active airway EMT, and beyond that to airway fibrosis and obstruction in COPD. Here we have provided the evidence for involvement of β-catenin and related transcription factors. Other potential drivers of EMT include the Notch and Hedgehog (Hh) pathways, while others have implicated the uPAR^53^ and cAMP systems as well. The relative importance of these pathways needs further investigation.

## Materials and Methods

### Ethics approval

The Tasmania Health & Medical Human Research Ethics Committee approved the study (EC00337, Chief Investigator, Sohal SS). All subjects gave written, informed consent prior to participation and all methods were performed in accordance with the relevant guidelines and regulations.

### Study Design

65 subjects were recruited through advertisement. Bronchial biopsies (BB) from 15 smokers with NLFS, 20 COPD-CS and 15 ex-smokers with COPD (COPD-EX) were compared with 15 healthy nonsmokers (NC) (Table 1). COPD was diagnosed according to Global Initiative for Chronic Obstructive Lung Disease (GOLD) criteria^54^. Subjects with other respiratory diseases, a history of recent acute exacerbation of COPD were excluded from the study. COPD subjects were on PRN (as needed) bronchodilators for the most part, and especially none were using systemic or inhaled corticosteroids.

**Table 1:** Demographic detail and lung function data for participants.

Groups (numbers)	NC (n = 15)	NLFS (n = 15)	COPD-CS (n = 20)	COPD-EX (n = 15)
GOLD I/GOLD II ^**‡**^	N/A	N/A	13/7	8/7
Male/female	7/8	11/4	12/8	9/6
Age (years)	44 (20–68) (p = 0.313)	50 (30–66) (p = 0.313)	61 (46–78) (p = 0.001)^*^	62 (53–69) (p = 0.001)^*^
Smoking (pack years)	0	32 (10–57)	45 (18–78)	51 (18–150)
FEV_1_% predicted (Post BD)^**†**^	113 (86–140)	99 (78–125) (p = 0.01)^*^	83 (66–102) (p < 0.001)^*^	83 (54–104) (p < 0.001)^*^
FEV_1_/FVC % (Post BD)^†^	82 (71–88)	77 (70–96) (p = 0.218)	59 (46–68) (p < 0.001)^*^	57 (38–68) p < 0.001)^*^

Data expressed as median and range. NC- Normal control; NLFS-Normal lung function smoker; COPD-CS-COPD current smoker; COPD-ES- COPD ex-smoker; N/A-Not any. ^*^Significance difference from NC. ^†^Post BD values after 400 µg of salbutamol. ^‡^Diagnosis of COPD was made according to GOLD 2015 guidelines.

### Tissue Processing and Immunostaining

Biopsies were first fixed in 10% neutral buffered formalin for 2 hrs, then in 50% ethanol before processing on a Leica ASP 200 tissue processor. Paraffin embedded sections of 3 mm were cut for staining, separated by at least 50 microns and mounted on slides. After removal of paraffin, sections were stained with either monoclonal antibody anti-β-catenin (abcam ab 22656 at 1/500 FOR 90 minutes at room temperature), monoclonal mouse anti-human E-cadherin (Dako, Clone NCH-38 at 1:30 for 40 minutes), rabbit polyclonal anti-Snail1 (abcam ab 180714 at 1/200 for 90 minutes at room temperature) after blocking with Dako serum block (X0909), following heat retrieval using a Dako PT link with high pH solution K800421 at 95 degrees for 15 minutes and Rabbit polyclonal anti-Twist (Abcam ab50581 at 1:500 for 90 minutes at room temperature) In each run a section stained with immunoglobulin (Ig) G1-negative control (X0931 clone DAKGO1; Dako Cytomation) was included to ensure absence of false positive staining and a known lung tissue positive tissue control was run with each staining. Bound antibodies were elaborated by using horseradish peroxidase (HRP) conjugated DAKO Envision plus reagent (cat no. K4001, anti-mouse or K4003 anti-rabbit) and diaminobenzidine (DAB) for a brown colour resolution (cat. no. K3468; Dako Cytomation). Nuclei were counterstained using Mayers Haematoxylin and sections dehydrated through ascending grades of ethanol, cleared in xylene and mounted in permount. We have extensively used and published with these methods^12, 33, 34, 55–60^.

### Tissue section analysis and quantitation

All slides were coded and randomized to blind the person who did the measurements (MM). We randomly choose five good fields for measurement from each slide, for each of the biomarkers. Only areas with intact epithelium and LP and without tissue damage were selected for measurement. Measurements were performed by computer-assisted image analysis using microscopy at 40× magnification (Leica DM 2500, Microsystems, Germany), a Spot insight 12 digital camera (Spot imaging, USA) and Image Pro V5.1 software (Media Cybernetics, USA).

All biomarkers (β-catenin, Snail and Twist) were quantitated as number basal and apical epithelial cells stained along with their differential percentages according to localization of antibody (cytoplasmic versus nuclear). Cells stained in the Rbm per mm of Rbm were treated in the same way, a were LP cells as differential percentage. We quantified the membranous staining of individual cells (basal, Rbm and LP) by counting as positive cells those with approximately 90% of the cell membrane positive. For cytoplasmic and nuclear staining, quantification was done according to area of each compartment (cell cytoplasm and nucleus) stained, for all cell with >20% of cytoplasmic/nuclear area stained and where cytoplasmic and nuclear staining could be differentiated from each other.

### Statistical analysis

Since the data were non-normally distributed, the results for each marker are presented as the median and range. Non-parametric ANOVA (Kruskal-Wallis) was first used to detect any overall difference among study groups, followed by Dunn’s multiple comparison test to specify which groups were different. Statistical analyses were performed using SPSS (statistics version 20.0, IBM Co, USA) for Windows 7.0 and a p-value of ≤0.05 was considered statistically significant.

## Conclusion and Summary

In conclusion, we have shown that the β-catenin-Snail1-Twist transcription factor cluster is activated in smokers and especially in COPD in epithelial basal cells, and that their expression is remarkably closely related to both EMT activity and airway obstruction. We feel that this work is opening novel understanding of the fundamental mechanisms involved in COPD patho-physiology.

## References

[1] Hogg JC (2004). Pathophysiology of airflow limitation in chronic obstructive pulmonary disease. Lancet.

[2] Barnes PJ (2003). New concepts in chronic obstructive pulmonary disease. Annu Rev Med.

[3] Johns DP, Walters JA, Walters EH (2014). Diagnosis and early detection of COPD using spirometry. J Thorac Dis.

[4] Sohal SS (2015). Chronic Obstructive Pulmonary Disease (COPD) and Lung Cancer: Epithelial Mesenchymal Transition (EMT), the Missing Link?. EBioMedicine.

[5] Yang IA (2011). Common pathogenic mechanisms and pathways in the development of COPD and lung cancer. Expert Opin Ther Targets.

[6] Sohal SS, Hansbro PM, Walters EH (2017). Epithelial Mesenchymal Transition in COPD, A Precursor for Epithelial Cancers: Understanding and Translation to Early Therapy. Annals of the American Thoracic Society.

[7] Sohal S (2010). Reticular basement membrane fragmentation and potential epithelial mesenchymal transition is exaggerated in the airways of smokers with chronic obstructive pulmonary disease. Respirology.

[8] Sohal S (2011). Evaluation of epithelial mesenchymal transition in patients with chronic obstructive pulmonary disease. Respir Res.

[9] Milara J, Peiro T, Serrano A, Cortijo J (2013). Epithelial to mesenchymal transition is increased in patients with COPD and induced by cigarette smoke. Thorax.

[10] Shaykhiev R, Crystal RG (2014). Early events in the pathogenesis of chronic obstructive pulmonary disease. Smoking-induced reprogramming of airway epithelial basal progenitor cells. Annals of the American Thoracic Society.

[11] Gohy ST (2015). Imprinting of the COPD airway epithelium for dedifferentiation and mesenchymal transition. Eur Respir J.

[12] Mahmood MQ (2017). Transforming growth factor (TGF) beta1 and Smad signalling pathways: A likely key to EMT-associated COPD pathogenesis. Respirology.

[13] Tian X (2011). E-cadherin/beta-catenin complex and the epithelial barrier. J Biomed Biotechnol.

[14] Pongracz JE, Stockley RA (2006). Wnt signalling in lung development and diseases. Respir Res.

[15] Eger A (2004). beta-Catenin and TGFbeta signalling cooperate to maintain a mesenchymal phenotype after FosER-induced epithelial to mesenchymal transition. Oncogene.

[16] Shin SY (2010). Functional roles of multiple feedback loops in extracellular signal-regulated kinase and Wnt signaling pathways that regulate epithelial-mesenchymal transition. Cancer Res.

[17] Heijink IH (2016). Cigarette smoke-induced epithelial expression of WNT-5B: implications for COPD. Eur Respir J.

[18] Fodde R, Brabletz T (2007). Wnt/beta-catenin signaling in cancer stemness and malignant behavior. Curr Opin Cell Biol.

[19] Hannigan G, Troussard AA, Dedhar S (2005). Integrin-linked kinase: a cancer therapeutic target unique among its ILK. Nat Rev Cancer.

[20] Medici D, Hay ED, Goodenough DA (2006). Cooperation between snail and LEF-1 transcription factors is essential for TGF-beta1-induced epithelial-mesenchymal transition. Mol Biol Cell.

[21] Nawshad A, Hay ED (2003). TGFbeta3 signaling activates transcription of the LEF1 gene to induce epithelial mesenchymal transformation during mouse palate development. J Cell Biol.

[22] Peinado H, Portillo F, Cano A (2004). Transcriptional regulation of cadherins during development and carcinogenesis. Int J Dev Biol.

[23] Yook JI (2006). A Wnt-Axin2-GSK3beta cascade regulates Snail1 activity in breast cancer cells. Nat Cell Biol.

[24] Vincent T (2009). A SNAIL1-SMAD3/4 transcriptional repressor complex promotes TGF-beta mediated epithelial-mesenchymal transition. Nat Cell Biol.

[25] Peinado H, Quintanilla M, Cano A (2003). Transforming growth factor beta-1 induces snail transcription factor in epithelial cell lines: mechanisms for epithelial mesenchymal transitions. J Biol Chem.

[26] Ansieau S (2008). Induction of EMT by twist proteins as a collateral effect of tumor-promoting inactivation of premature senescence. Cancer Cell.

[27] Xu J, Lamouille S, Derynck R (2009). TGF-beta-induced epithelial to mesenchymal transition. Cell Res.

[28] Wang J (2007). Opposing LSD1 complexes function in developmental gene activation and repression programmes. Nature.

[29] Oldenburger. A. *et al*. A-kinase anchoring proteins contribute to loss of E-cadherin and bronchial epithelial barrier by cigarette smoke. *Am J Physiol Cell Physiol* **306**, doi:10.1152/ajpcell.00183.2013 (2014). 10.1152/ajpcell.00183.2013 24452374

[30] Hogg JC, McDonough JE, Suzuki M (2013). Small airway obstruction in COPD: new insights based on micro-CT imaging and MRI imaging. Chest.

[31] Dunnill MS (1969). The classification and quantification of emphysema. Proc R Soc Med.

[32] Prudkin L (2009). Epithelial-to-mesenchymal transition in the development and progression of adenocarcinoma and squamous cell carcinoma of the lung. Mod Pathol.

[33] Mahmood MQ, Ward C, Muller HK, Sohal SS, Walters EH (2017). Epithelial mesenchymal transition (EMT) and non-small cell lung cancer (NSCLC): a mutual association with airway disease. Medical oncology (Northwood, London, England).

[34] Mahmood MQ (2015). Epithelial mesenchymal transition in smokers: large versus small airways and relation to airflow obstruction. Int J Chron Obstruct Pulmon Dis.

[35] Ryan DM (2014). Smoking dysregulates the human airway basal cell transcriptome at COPD risk locus 19q13.2. PLoS One.

[36] Nowrin, K., Sohal, S. S., Peterson, G., Patel, R. & Walters, E. H. Epithelial-mesenchymal transition as a fundamental underlying pathogenic process in COPD airways: fibrosis, remodeling and cancer. *Expert Rev Respir Med*, 1–13, doi:10.1586/17476348.2014.948853 (2014). 10.1586/17476348.2014.948853 25113142

[37] Jolly, M. K. *et al*. Epithelial-mesenchymal transition, a spectrum of states: Role in lung development, homeostasis, and disease. *Dev Dyn*, doi:10.1002/dvdy.24541 (2017). 10.1002/dvdy.24541 28646553

[38] Cheon SS, Nadesan P, Poon R, Alman BA (2004). Growth factors regulate β-catenin-mediated TCF-dependent transcriptional activation in fibroblasts during the proliferative phase of wound healing. Experimental cell research.

[39] Chilosi M (2003). Aberrant Wnt/β-catenin pathway activation in idiopathic pulmonary fibrosis. The American journal of pathology.

[40] Clevers H (2006). Wnt/β-catenin signaling in development and disease. Cell.

[41] Nunes RO (2008). GSK-3/β-catenin signaling axis in airway smooth muscle: role in mitogenic signaling. Am J Physiol-Lung C.

[42] Guo X, Wang X-F (2009). Signaling cross-talk between TGF-β/BMP and other pathways. Cell research.

[43] Clifford RL, Deacon K, Knox AJ (2008). Novel Regulation of Vascular Endothelial Growth Factor-A (VEGF-A) by Transforming Growth Factor β1 REQUIREMENT FOR Smads, β-CATENIN, AND GSK3β. Journal of Biological Chemistry.

[44] Doyle JL, Haas TL (2009). Differential role of β‐catenin in VEGF and histamine‐induced MMP‐2 production in microvascular endothelial cells. Journal of cellular biochemistry.

[45] Rahmani M (2005). Regulation of the versican promoter by the β-catenin-T-cell factor complex in vascular smooth muscle cells. Journal of Biological Chemistry.

[46] Nagarajan D, Melo T, Deng Z, Almeida C, Zhao W (2012). ERK/GSK3β/Snail signaling mediates radiation-induced alveolar epithelial-to-mesenchymal transition. Free Radical Biology and Medicine.

[47] Xiong, S. *et al*. Regulatory T Cells Promote β-Catenin–Mediated Epithelium-to-Mesenchyme Transition During Radiation-Induced Pulmonary Fibrosis. *International Journal of Radiation Oncology* • *Biology* • *Physics* **93**, 425–435, doi:10.1016/j.ijrobp.2015.05.043. 10.1016/j.ijrobp.2015.05.043 26253394

[48] Baarsma HA (2011). Activation of WNT/β-catenin signaling in pulmonary fibroblasts by TGF-β 1 is increased in chronic obstructive pulmonary disease. PLoS One.

[49] Nishioka M (2015). Fibroblast-epithelial cell interactions drive epithelial-mesenchymal transition differently in cells from normal and COPD patients. Respiratory Research.

[50] Morin PJ (1999). β‐catenin signaling and cancer. Bioessays.

[51] Li J, Zhou BP (2011). Activation of beta-catenin and Akt pathways by Twist are critical for the maintenance of EMT associated cancer stem cell-like characters. BMC cancer.

[52] Pozharskaya V (2009). Twist: a regulator of epithelial-mesenchymal transition in lung fibrosis. PLoS One.

[53] Wang Q, Wang Y, Zhang Y, Zhang Y, Xiao W (2013). The role of uPAR in epithelial-mesenchymal transition in small airway epithelium of patients with chronic obstructive pulmonary disease. Respir Res.

[54] GOLD. Global Strategy for the Diagnosis, Mangement and Prevention of Chronic Obstructive Pulmonary Disease.Update 2007 (2007).

[55] Eapen, M. S. *et al*. Profiling cellular and inflammatory changes in the airway wall of mild to moderate COPD. *Respirology*, doi:10.1111/resp.13021 (2017). 10.1111/resp.13021 28326668

[56] Sohal, S. S. *et al*. Reticular basement membrane fragmentation and potential epithelial mesenchymal transition is exaggerated in the airways of smokers with chronic obstructive pulmonary disease. *Respirology* **15**, doi:10.1111/j.1440-1843.2010.01808.x (2010). 10.1111/j.1440-1843.2010.01808.x 20630030

[57] Sohal SS (2011). Evaluation of epithelial mesenchymal transition in patients with chronic obstructive pulmonary disease. Respir Res.

[58] Sohal SS (2013). Changes in Airway Histone Deacetylase2 in Smokers and COPD with Inhaled Corticosteroids: A Randomized Controlled Trial. PLoS One.

[59] Sohal SS (2014). A randomized controlled trial of inhaled corticosteroids (ICS) on markers of epithelial-mesenchymal transition (EMT) in large airway samples in COPD: an exploratory proof of concept study. Int J Chron Obstruct Pulmon Dis.

[60] Soltani A (2016). Inhaled corticosteroid normalizes some but not all airway vascular remodeling in COPD. Int J Chron Obstruct Pulmon Dis.

